# Direct comparison of magnetic resonance imaging and pathological shrinkage patterns of triple-negative breast cancer after neoadjuvant chemotherapy

**DOI:** 10.1186/s12957-020-01959-9

**Published:** 2020-07-21

**Authors:** Katsuhiro Yoshikawa, Mitsuaki Ishida, Naoki Kan, Hirotsugu Yanai, Koji Tsuta, Mitsugu Sekimoto, Tomoharu Sugie

**Affiliations:** 1 Department of Pathology and Clinical Laboratory, Kansai Medical University, 2-5-1, Shinmachi, Hirakata City, Osaka Prefecture 573-1010 Japan; 2 Department of Surgery, Kansai Medical University, 2-5-1, Shinmachi, Hirakata City, Osaka Prefecture Japan; 3 Department of Radiology, Kansai Medical University, 2-5-1, Shinmachi, Hirakata City, Osaka Prefecture Japan

**Keywords:** Triple-negative breast cancer, Magnetic resonance imaging, Neoadjuvant chemotherapy, Residual tumor diameter, Predictive equation

## Abstract

**Background:**

We aimed to investigate the usefulness of magnetic resonance imaging (MRI) and histopathological shrinkage patterns to formulate a predictive equation for estimating residual tumor size after neoadjuvant chemotherapy (NAC) in triple-negative breast cancer (TNBC) patients.

**Methods:**

We enrolled 34 TNBC patients who underwent MRI before and after NAC. The MRI and histopathological shrinkage patterns were analyzed and classified into five categories—types I and II (concentric shrinkage without or with surrounding lesions, respectively), type III (shrinkage with residual multinodular lesions), type IV (diffuse contrast enhancement in the entire quadrant), and non-visualization. The residual tumor sizes following MRI and histopathological examination were also compared.

**Results:**

The most common MRI and histopathological shrinkage pattern was type I (41.2% and 38.2%, respectively), followed by non-visualization (26.5% and 32.4%, respectively); the concordance rate between MRI and histopathological shrinkage patterns was 41.2%. There was a strong correlation between MRI tumor size and pathological tumor size (*r* = 0.89). Based on these findings, a predictive equation for pathological tumor size was formulated as follows: pathological tumor size (mm) = 1.1502 × (MRI tumor size [mm]) + 8.4277.

**Conclusions:**

Our equation may aid accurate preoperative assessment. Further studies are needed to determine its predictive value and applicability.

## Background

Triple-negative breast cancer (TNBC), defined by a lack of estrogen receptor, progesterone receptor, and human epidermal growth factor receptor 2 (HER2) expression, is a high-grade phenotype of breast cancer with a poor prognosis [1, 2]. Although TNBC represents 12–17% of breast cancer patients, it is well-known that the rates of recurrence and/or distant metastasis, as well as mortality, are significantly higher in TNBC than other breast cancer subtypes [3]. Patients with TNBC therefore often require adjuvant chemotherapy. Several large randomized clinical trials have demonstrated equivalent efficacy between neoadjuvant chemotherapy (NAC) and adjuvant chemotherapy and suggest that NAC improves the success rate of breast-conserving surgery (BCS) [4–9]. Therefore, NAC is particularly recommended for patients with TNBC who are intended to receive BCS [10]. However, because patients undergoing NAC have higher local recurrence rates, the extent of resection must be carefully determined [11].

Magnetic resonance imaging (MRI) is widely used and plays a pivotal role in guiding the extent of breast surgery by providing estimates of the tumor size and distribution after NAC [12, 13]. The superiority of MRI over ultrasound and mammography is well-recognized [14]. Obtaining accurate imaging information on the extent and distribution of residual carcinoma after NAC is of utmost importance, as over-estimation of the residual tumor size may lead to unnecessary mastectomies. Conversely, under-estimation may result in tumors on ink followed by additional surgeries. Despite this, there are very few reports comparing MRI and histopathology in terms of shrinkage patterns and tumor size. Our study intended to analyze the detailed radiopathological correlation between MRI patterns before and after NAC and pathological tumor residual patterns in patients with TNBC. The tumor diameter after NAC was also evaluated using MRI, and a predictive equation was developed to estimate the residual tumor diameter based on these findings.

## Methods

### Patient selection

We enrolled 165 consecutive patients with TNBC who underwent surgical resection at the Department of Surgery of the Kansai Medical University Hospital between January 2006 and December 2018. Patients who did not receive NAC or did not undergo MRI before and after NAC were excluded from the study; finally, 34 patients with TNBC were included.

This study was conducted in accordance with the principles of the Declaration of Helsinki, and the study protocol was approved by the Institutional Review Board of the Kansai Medical University Hospital (protocol no. 2019041). Informed consent was individually obtained from all participants included in the study.

### Chemotherapy

The NAC regimens were selected based on patients’ preferences. Overall, 33 (97%) patients received sequential anthracycline- and taxane-based regimens. The anthracycline-based regimens included EC (epirubicin 100 mg/m^2^ and cyclophosphamide 500 mg/m^2^), AC (doxorubicin 60 mg/m^2^ and cyclophosphamide 600 mg/m^2^), and FEC (epirubicin 100 mg/m^2^, cyclophosphamide 500 mg/m^2^, and 5-fluorouracil 500 mg/m^2^). Chemotherapy was administered every 3 weeks for 4 cycles. The taxane-based regimens included docetaxel at a dose of 70 mg/m^2^ every 3 weeks for 4 cycles, or weekly paclitaxel at a dose of 80 mg/m^2^ for 12 doses with scheduled rests. Only one patient received a taxane-based regimen without anthracyclines (docetaxel 75 mg/m^2^ and cyclophosphamide 600 mg/m^2^).

### MRI technique

MRI was performed using 1.5-T scanners (Signa Excite HD; GE Healthcare, Milwaukee, WI, USA) with a dedicated breast coil. All images were obtained in the prone position. All patients received intravenous contrast (0.1 mmol/kg gadopentetate dimeglumine).

MRI was performed 2 to 4 weeks prior to and following completion of NAC.

### Interpretation of MRI

Two experienced breast surgeons and one experienced breast-imaging radiologist conjointly reviewed the pre- and post-NAC breast MRI findings of all patients.

The contrast-enhanced MRI patterns prior to NAC were classified into five categories, as described by Tomida et al. [15]—solitary, grouped (localized lesion with linear and/or spotty enhancement), separated (multiple foci of contrast enhancement), mixed (grouped lesion with multiple foci), and replaced (diffuse contrast enhancement in entire quadrant) lesions. The MRI shrinkage patterns of breast cancer following NAC were classified into five categories, as suggested by Kim et al. [16]—type I (concentric shrinkage without any surrounding lesion), type II (concentric shrinkage with surrounding lesions), type III (shrinkage with residual multi-nodular lesions), type IV (diffuse contrast enhancement in entire quadrant), and non-visualization. Breast cancer reactions after NAC were determined using the response evaluation criteria in solid tumor classification [17], based on unidimensional measurements of the largest tumor diameter.

### Histopathological examination

The histopathological diagnosis of the breast cancers was independently performed by two experienced diagnostic pathologists. The response following NAC was assessed based on the Miller–Payne grading system, established by Ogston et al. [18]. The grades are as follows: grade 1, no change or particular alteration to individual malignant cells and no reduction in overall cellularity; grade 2, minor loss of tumor cells (≤ 30%) with overall cellularity remaining high; grade 3, an estimated 30 to 90% reduction in tumor cells; grade 4, a marked disappearance of tumor cells so that only small clusters or widely dispersed individual cells remain, with > 90% loss of tumor cells; and grade 5, no malignant cells identified in sections from the tumor site, only vascular fibroelastic stroma remaining, often containing macrophages, with possible presence of intraductal carcinoma components.

The histopathological patterns of the residual tumor were also classified into five categories, similar to the classification of MRI shrinkage patterns [15]. Histopathological tumor size was determined based on unidimensional measurements of the largest tumor diameter (including ductal component). A free margin of 2 mm or less was defined as positive [19].

### Statistical analysis

All analyses were performed using the SPSS Statistics 25.0 software (IBM, Armonk, NY, USA). Agreement between two groups was analyzed using the Kappa test.

The Pearson correlation coefficient was used to compare tumor size on MRI with the histological tumor size. A *P* value of less than 0.05 was regarded as significant. Moreover, a predictive equation for the pathological tumor size based on MRI findings was evaluated using the least square method.

## Results

### Patients’ characteristics

The characteristics of patients in the present cohort are summarized in Table 1. This study included 34 patients; all were female. The median age at the time of initial diagnosis was 53 (range 31–77) years. All patients were diagnosed with TNBC based on the biopsy specimens; 31, 2, and 1 patients were diagnosed with invasive carcinoma of no special type, apocrine carcinoma, and invasive lobular carcinoma, respectively. Ten patients were initially diagnosed with clinical stage I, 10 with stage IIA, 9 with stage IIB, 2 with stage IIIA, 1 with stage IIIB, and 2 with stage IIIC, respectively, according to the 8th Union for International Cancer Control TNM Classification [20].

**Table 1:** Clinical characteristics of patients with triple negative breast cancer

Factor	Median (range)	*n*	%
Total		34	
Age (years)	53 (31–77)		
Menopausal status	Premenopausal	11	32.4
	Postmenopausal	20	58.8
	Unknown	3	8.8
Histology	Invasive carcinoma, no special type	31	91.2
	Invasive lobular carcinoma	1	2.9
	Apocrine carcinoma	2	5.9
Initial clinical stage	I	10	29.4
	IIA	10	29.4
	IIB	9	26.5
	IIIA	2	5.9
	IIIB	1	2.9
	IIIC	2	5.9
Ki-67	Low (≤ 20%)	2	5.9
	High (> 20%)	29	85.3
	Unknown	3	8.8
Lymph node metastasis	Present	15	44.1
	Absent	19	55.9
Neoadjuvant chemotherapy regimen	Taxane	1	2.9
	Taxane + anthracycline	33	97.1
Type of surgery	Radical mastectomy	11	32.4
	Partial mastectomy	23	67.6

### MRI findings before and after NAC

**Fig. 1:**
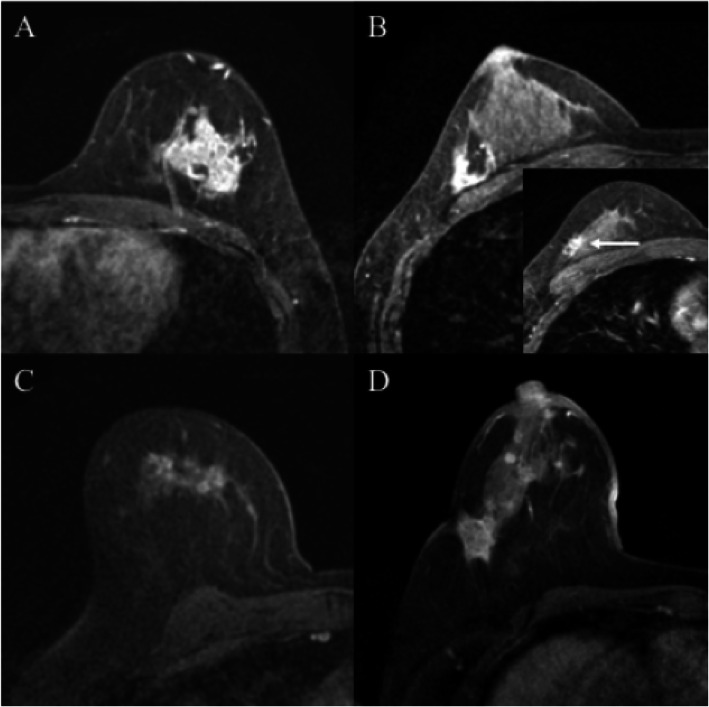
Magnetic resonance images of the initial enhancement patterns of triple-negative breast cancer prior to neoadjuvant chemotherapy. **a** Solitary, **b** separated (arrow shows the daughter nodule), **c** grouped, and **d** replaced

The most common enhancement pattern before NAC was solitary (20 patients, 58.8%), followed in order by separated (7 patients, 20.6%), grouped (5 patients, 14.7%), and replaced (2 patients, 5.9%) lesions (Fig. 1a–d; Table 2). None of the patients in the present study showed mixed enhancement patterns.

**Table 2:** Correlation between initial contrast-enhancement patterns prior to neoadjuvant chemotherapy and shrinkage patterns

Shrinkage patterns	Initial enhancement patterns			
	Solitary	Grouped	Separated	Replaced
Non-visualization	6	0	3	0
Type I	12	1	1	0
Type II	1	2	1	0
Type III	1	2	2	0
Type IV	0	0	0	2

**Fig. 2:**
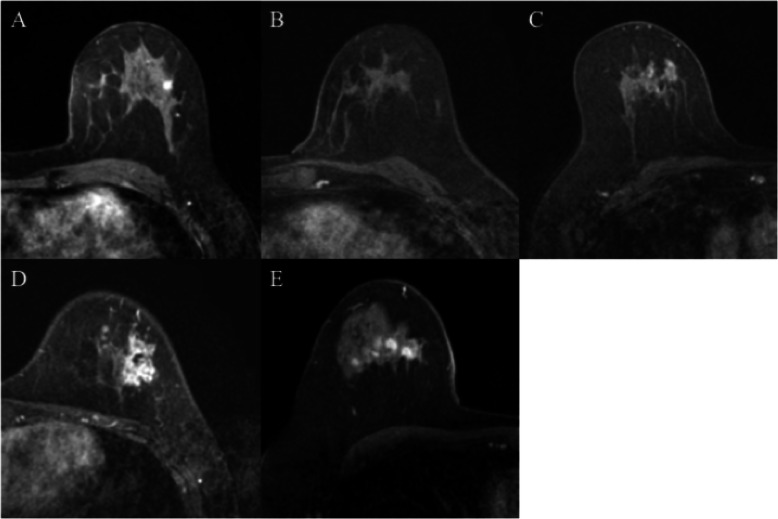
Magnetic resonance images of the shrinkage patterns of triple-negative breast cancer following neoadjuvant chemotherapy. **a** Concentric shrinkage without any surrounding lesions, type I; **b** non-visualization; **c** residual multinodular lesions, type III; **d** concentric shrinkage pattern with surrounding lesions, type II; **e** diffuse contrast enhancement in entire quadrant, type IV

Table 2 summarizes the tumor shrinkage patterns after NAC. The most common shrinkage pattern on MRI was type I (14 patients, 41.2%), followed in order by non-visualization (9 patients, 26.5%), type III (5 patients, 14.7%), type II (4 patients, 11.8%), and type IV (2 patients, 5.9%) (Fig. 2a–e; Table 2). For the association between the initial findings and shrinkage patterns in MRI, the most common shrinkage pattern (type I) was observed in 60% of the patients with solitary patterns, followed by non-visualization, which was observed in 30% (Table 2). Patients with non-visualization patterns after NAC initially had either solitary or separated enhancement patterns. MRI shrinkage patterns did not significantly correlate with the clinicopathological parameters, including the menopausal status, clinical stage of the tumor, and Ki-67 labeling index.

### Histopathological features following NAC

**Fig. 3:**
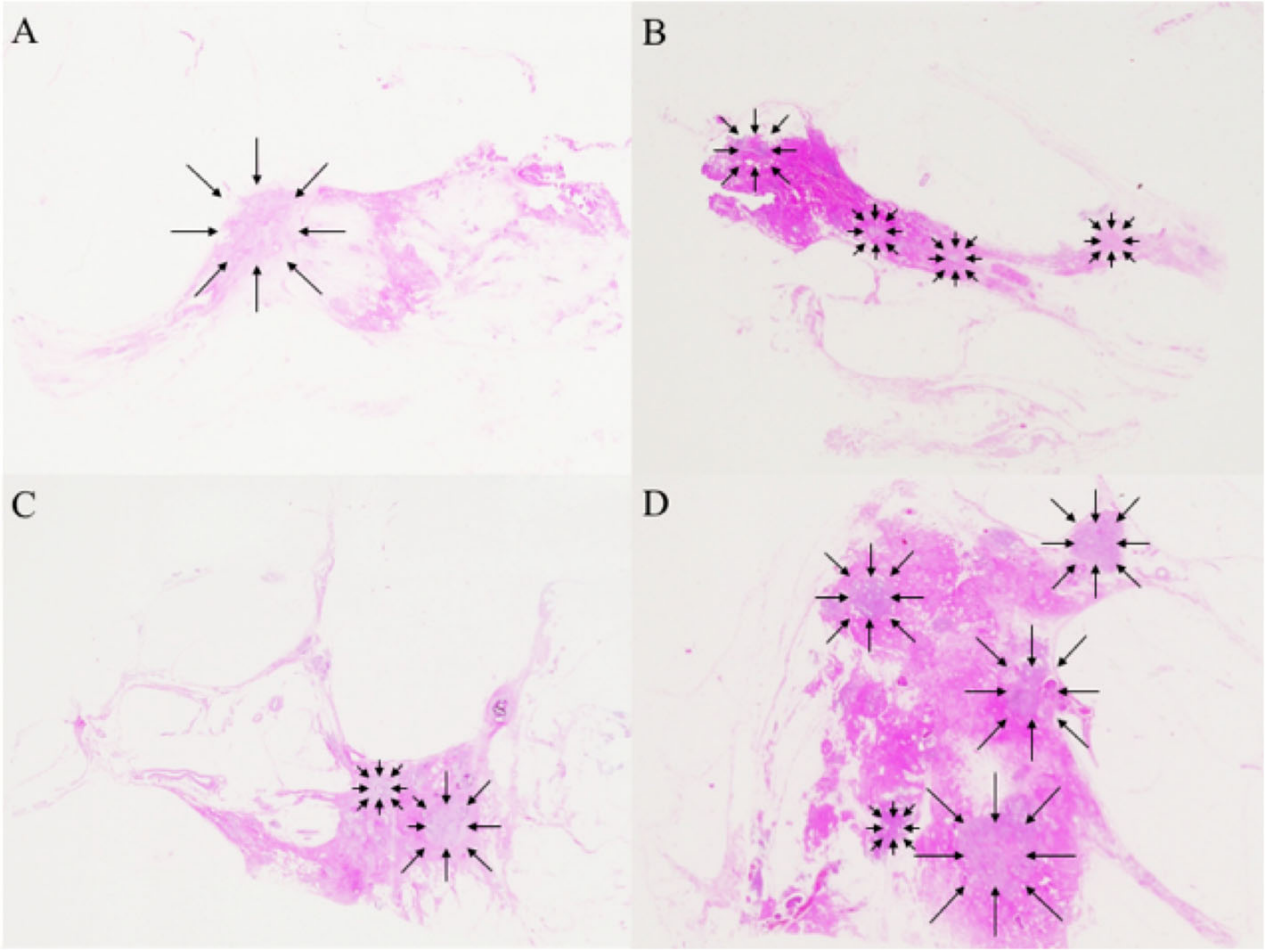
Panoramic view of the histopathological shrinkage patterns and features following neoadjuvant chemotherapy. **a** Pathological type I showing concentric shrinkage without any surrounding lesions. **b** Pathological type III demonstrating shrinkage with residual multinodular lesions. **c** Pathological type II showing concentric shrinkage with surrounding lesions. **d** Pathological type IV demonstrating multiple residual carcinomas (arrows: residual carcinoma cells); hematoxylin and eosin staining

The most common histopathological regression grade was 1 (12 patients, 35.3%), followed by grades 5 (11 patients, 32.4%), 2 (6 patients, 17.6%), 4 (4 patients, 11.8%), and 3 (1 patient, 2.9%) (Fig. 3a–d; Table 3). The most common shrinkage pattern on histopathology was type I, observed in 38.2% of the patients, followed in order by non-visualization (32.1%), type III (20.6%), type II (5.9%), and type IV (2.9%) (Table 3). The observed concordance between the shrinkage patterns on MRI and on histopathology was 41.2% (Kappa statistics 0.181, *p =* 0.07). Subsequently, we performed a direct comparison between MRI-based and histopathological shrinkage patterns in TNBC. Among 11 patients with no residual disease, six were diagnosed as having non-visualization pattern on MRI (sensitivity, 54.5%). In the other five patients, type I MRI shrinkage pattern was observed in 1 patient, type II in 2, and type III in 2 other patients (Table 3). Among the 23 patients who had residual disease, three did not show contrast-enhanced lesions on MRI (specificity 87.0%). Pathological shrinkage patterns did not significantly correlate with the clinicopathological parameters, including the menopausal status, clinical stage of the tumor, and Ki-67 labeling index.

**Table 3:** Correlation between MRI shrinkage patterns and pathological shrinkage patterns following neoadjuvant chemotherapy

	Pathological shrinkage pattern				
	Non-visualization	Type I	Type II	Type III	Type IV
MRI shrinkage pattern					
Non-visualization	6	2	0	1	0
Type I	1	8	1	3	1
Type II	2	0	0	2	0
Type III	2	2	1	0	0
Type IV	0	1	0	1	0

### Relationship between MRI and histopathological tumor sizes after NAC

**Fig. 4:**
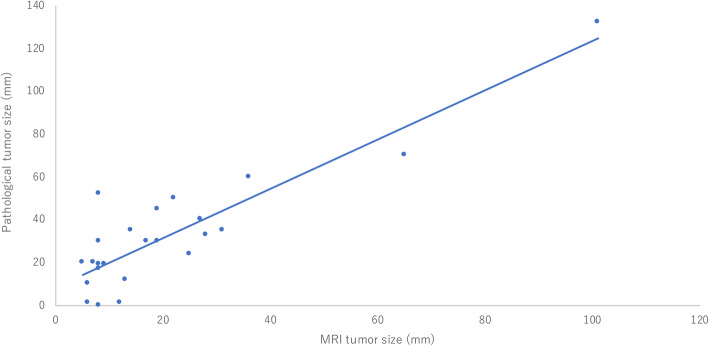
Correlation between MRI and pathological tumor sizes in patients with non-clinical complete remission. Correlation coefficient (*r*) = 0.89 (*p* < 0.0001)

Next, we assessed the residual tumor size on MRI and on histopathology after NAC in 25 patients with TNBC. There was a significant correlation between MRI tumor size and histopathological tumor size (Pearson’s correlation coefficient of 0.89, *p* < 0.0001) (Fig. 4). The mean difference between MRI-based and histopathology-based tumor diameter was 11.5 ± 12.7 mm. The following equation was developed based on the findings:

$$ \mathrm{Pathological}\ \mathrm{residual}\ \mathrm{tumor}\ \mathrm{size}\ \left(\mathrm{mm}\right)=1.1502\times \left(\mathrm{residual}\ \mathrm{tumor}\ \mathrm{size}\ \left[\mathrm{mm}\right]\right)+8.4277 $$

This equation can be applied to patients with MRI shrinkage patterns that are reflective of types I, II, III, and IV (excluding non-visualization). “Pathological residual tumor size” implies the total tumor size including invasive carcinoma and intraductal carcinoma components. If a resection size was estimated based on this equation before surgery, a safe margin of 10 mm beyond the calculated pathological size would lead to a positive margin rate of 4%. Moreover, a 20-mm safe margin would result in a positive margin rate of 0%.

## Discussion

In the present study, we demonstrated a significant correlation between MRI and histopathology for residual tumor size and obtained a predictive equation to estimate the size of residual tumors using MRI results. Furthermore, the correlation between MRI and histopathological shrinkage patterns in patients with TNBC after NAC was relatively high.

Several reports have elucidated the shrinkage patterns of breast cancer after NAC. Kim et al. studied the shrinkage tumor patterns on MRI following NAC, and they reported that the most common MRI shrinkage patterns in breast cancer patients were type I (51.8%), followed by type II (23.2%) [16]. Tomida et al. studied the correlation between MRI shrinkage patterns and pathological shrinkage patterns following NAC. They reported that the most common pathological shrinkage pattern was type II (40.7%), followed by type III (29.6%), and the concordance rate between MRI and histopathological patterns was 48% [15]. However, these two studies did not examine the relationship between the shrinkage patterns of various molecular subtypes of breast cancer. In the present study, the high rate of type I (41.2%), the relatively high rate of non-visualization (26.5%), and the low rate of type II (11.8%) patterns may be a characteristic feature of MRI shrinkage patterns in TNBC. Furthermore, the present study found that high frequencies of type I (38.2%) and non-visualization (32.4%) pathological shrinkage patterns were characteristic of TNBC. The concordance rate of 41.2% between MRI and pathological shrinkage patterns in the present cohort was comparable to that of the previous report (48%), in which no distinction among molecular subtypes was found [15].

Because of the high effectiveness of chemotherapy observed in patients with TNBC [21], our study resulted in a high frequency of the “non-visualization” pathological pattern, compared to previous reports [15, 16]. Moreover, the high frequency of type I pathological shrinkage pattern suggests that the concentric shrinkage pattern may be more commonly found in TNBC than in other molecular subtypes after NAC. These characteristics of TNBC may be attributed to differences in the efficacy of NAC, evidenced by the variation observed in the tumor shrinkage patterns after NAC among different molecular subtypes.

MRI is known to be useful for predicting pathological complete responses (pCR) after NAC. Liu et al. reported a high specificity (88%) and relatively low sensitivity (65%) for MRI in predicting pCR among patients with breast cancer [22]. Although the molecular subtypes were not classified in their reports, the results of the present study concur with theirs. Therefore, TNBC may have a similar tendency to other molecular subtypes on pCR prediction.

Nakahara et al. reported that TNBC showed a significant correlation between the residual tumor sizes obtained on MRI and pathological tumor sizes after NAC [23]. However, an MRI-based predictive equation for residual tumor size has yet to be developed. The development of an accurate MRI-based measurement tool is of particular necessity for patients receiving NAC and BCS, a matter that needs to be urgently addressed. Our predictive equation may help to estimate accurately tumor size and to reduce the high rate (2–40%) of tumors on ink reported in patients undergoing BCS after NAC [24].

The present study had several limitations. First, it was a retrospective single-institution study with a small sample size, and therefore, selection bias was a possibility. Second, the present study only analyzed TNBC. Since the MRI and pathological shrinkage patterns after NAC may be influenced by molecular subtypes, further studies are needed to investigate the characteristics of residual carcinoma after NAC in patients with various molecular types of breast cancer. Third, for our predictive equation, a validation study is required before it can be formally used; therefore, additional studies are needed to evaluate its applicability.

## Conclusions

In the present study, we investigated the characteristics of MRI and pathological shrinkage patterns and their relationship in patients with TNBC. We also developed a useful equation for estimating the size of residual cancer after NAC in patients with TNBC, based on the size noted on MRI. The newly developed equation may improve the success rates for BCS after NAC among patients with TNBC.

## Data Availability

The datasets used and/or analyzed during the current study are available from the corresponding author on reasonable request.

## Abbreviations

BCS: Breast-conserving surgery
HER2: Human epidermal growth factor receptor 2
MRI: Magnetic resonance imaging
NAC: Neoadjuvant chemotherapy
pCR: Pathological complete responses
TNBC: Triple-negative breast cancer
